# Genetic Dissection of Resistance to Northern Corn Leaf Blight in a Large Commercial Maize Hybrid Population

**DOI:** 10.3390/ijms27114983

**Published:** 2026-05-30

**Authors:** Wei Chen, Yishuo Niu, Rui Han, Yongzhen Yu, Jie Zhang, Haipeng Yang, Yafei Liu, Pengjia Bu, Lin Li, Hongwei Zhang

**Affiliations:** 1 Maize Research Institute, Shanxi Agricultural University, Xinzhou 034000, China; 2 National Key Laboratory of Crop Genetic Improvement, Huazhong Agricultural University, Wuhan 430070, China; 13971434942@139.com (Y.N.);; 3 Hubei Hongshan Laboratory, Wuhan 430070, China; 4 State Key Laboratory of Crop Gene Resources and Breeding, National Key Facility for Crop Gene Resources and Genetic Improvement, Institute of Crop Sciences, Chinese Academy of Agricultural Sciences, Beijing 100081, China

**Keywords:** northern corn leaf blight, commercial maize hybrids, genome-wide association study

## Abstract

Northern corn leaf blight (NCLB), caused by *Setosphaeria turcica*, is a major foliar disease of maize. To dissect the genetic basis of NCLB resistance in breeding-relevant germplasm, we evaluated NCLB severity in a panel of 500 commercial maize hybrids under natural disease pressure over a single growing season. High-throughput genotyping generated densely distributed SNP markers. The stratification of the panel into four major genetic subgroups, together with the relatively rapid LD decay (approximately 84.9 kb), indicated abundant genetic variation and favorable mapping resolution in the hybrid population. Phenotypic analysis revealed that most hybrids showed resistance or moderate resistance to NCLB. The broad-sense heritability of NCLB severity was estimated at 0.6588. Genome-wide association studies using the FarmCPU, BLINK, and MLMM models identified 17 consensus association signals, and four candidate genes were prioritized, including *ZmCW1*, *CALS2*, *ZmCW2*, and *NCED8*. A candidate gene-centered network identified 27 direct connections related to *ZmCW1*, *CALS2*, and *ZmCW2*. GO enrichment analysis showed that genes in these networks may regulate NCLB resistance through oxidative stress and redox-related processes. These results unravel the genetic architecture underlying NCLB resistance in commercial maize hybrids and nominate target loci for maize resistance breeding.

## 1. Introduction

Northern corn leaf blight (NCLB) remains one of the most important foliar diseases of maize worldwide [1,2]. The disease is caused by the heterothallic ascomycete *Setosphaeria turcica* (anamorph: *Exserohilum turcicum*). It occurs in tropical, subtropical, and temperate production regions worldwide [3,4]. Under humid conditions, epidemics typically begin on lower leaves and spread upward, reducing functional leaf area and photosynthetic capacity during critical development stages [1,3]. Yield loss can be severe when infection develops before or around silking [5,6]. Because of its broad distribution and recurrent epidemics, NCLB remains an important target of maize disease-resistance breeding.

The durability of NCLB resistance is closely related to pathogen population biology and virulence evolution [1,3,7]. Virulence diversity in *S. turcica* is commonly described in terms of physiological races defined by differential interactions with *Ht* (*Helminthosporium turcicum*) resistance genes, a group of resistance (R) genes in maize that specifically defend against NCLB [1,3]. Surveys conducted in different maize-growing regions of China have revealed complex race compositions, indicating ongoing pathogen evolution [8,9]. The detection of both *S. turcica* mating types, MAT1-1 and MAT1-2, in multiple production areas further suggests the potential for sexual recombination and substantial gene flow [9,10]. Therefore, the identification of durable host-resistance sources remains essential for effective NCLB management [1,3].

The genetic basis of NCLB resistance has been investigated mainly through biparental QTL mapping and genome-wide association studies (GWAS) [5,11]. Compared with biparental populations, GWAS in diverse germplasm can exploit historical recombination to improve mapping resolution and broaden allelic sampling [11,12]. Recent studies using high-density markers and multi-locus models have identified multiple loci associated with NCLB response [2,4]. However, inbred-based GWAS cannot fully represent the genetic basis of traits expressed in commercial hybrids, where heterosis, dominance effects, and epistatic interactions are prevalent. Loci identified in inbred lines often show inconsistent effects when transferred into hybrid breeding programs, limiting their direct application in practical maize improvement [13,14]. Despite the importance of hybrid performance in production, GWAS of commercial hybrids remains limited, leading to a gap between genetic mapping findings and breeding needs.

In this study, we combined high-density genotyping, field phenotyping, and population genetic analysis to investigate NCLB severity under natural disease pressure. We obtained plot-level disease phenotypes and performed genotype-based population analyses. By performing multi-model GWAS and single-locus genotype comparison in this hybrid population, we aimed to identify loci, candidate genes, and their regulatory relationships in maize hybrids. The novelties of this study are: first, it provides empirical evidence for the feasibility of hybrid-based GWAS in dissecting maize disease; second, it identifies a set of stable loci and candidate genes that may be useful for commercial hybrid breeding. These findings not only enrich our understanding of the genetic architecture of NCLB resistance but also establish an efficient strategy for improving hybrid performance.

## 2. Results

### 2.1. Genetic Structure Analysis of the Hybrid Population

**Figure 1:**
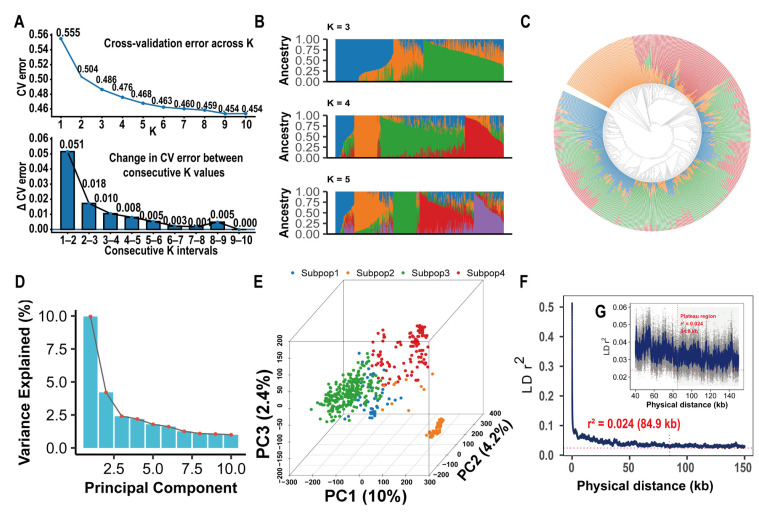
Four major genetic groups were resolved in the commercial hybrid panel. (**A**) Cross-validation error declined continuously with increasing K. (**B**) Comparison of subpopulation division with K = 3, 4, and 5. (**C**) The neighbor-joining tree separated the panel into four major branches. (**D**,**E**) Principal component analysis resolved four partially overlapping clusters, with PC1 and PC2 explaining 10.0% and 4.2% of the total genetic variation, respectively. (**F**) The genome-wide LD decay status. (**G**) Close-up view of the LD decay at approximately 89.4 kb.

Genome-wide analyses indicated that the commercial hybrid panel is genetically diverse and structured. In ADMIXTURE analysis, the change in CV error between consecutive K values became progressively smaller, and the decrease from K = 4 to K = 5 was already less than 0.01 (ΔCV error = 0.00802; Figure 1A), indicating that the major population subdivision had largely been captured by K = 4. Consistent with this interpretation, the ancestry bar plots showed that K = 4 more clearly resolved four major ancestral components (Figure 1B). The neighbor-joining tree separated the panel into three major branches (Figure 1C). PC1 and PC2 explained 10.0% and 4.2% of the total genetic variation, respectively (Figure 1D), and the PCs were also broadly consistent with the four-group pattern (Figure 1E). Genome-wide linkage disequilibrium (LD) decayed rapidly, reaching approximately 84.9 kb at r^2^ = 0.024 (Figure 1F), indicating that the panel retained high mapping resolution for association analysis. Subgroup assignment and field phenotype for each coded hybrid are provided in Supplementary Table S1.

### 2.2. Extensive Phenotypic Variation and High Heritability of NCLB Resistance

**Figure 2:**
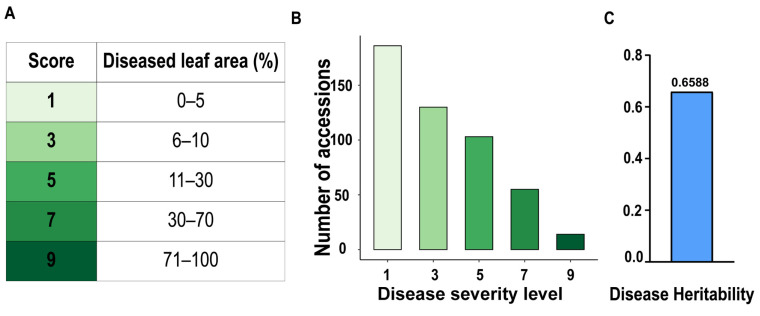
NCLB severity varied widely across the commercial hybrid panel. (**A**) NCLB severity was scored on a five-level scale based on the proportion of diseased leaf area. Scores 1, 3, 5, 7, and 9 correspond to 0–5%, 6–10%, 11–30%, 30–70%, and 71–100%, respectively. (**B**) Among the 488 hybrids with valid phenotypes, 186, 130, 103, 55, and 14 entries were assigned to scores 1, 3, 5, 7, and 9, respectively. (**C**) The broad-sense heritability of NCLB severity.

Field phenotyping captured substantial variation in NCLB response across the commercial hybrid panel. Figure 2A shows representative leaf symptoms and the rating scheme used for disease scoring, with five NCLB severity classes defined according to the proportion of diseased leaf area. On this basis, the 488 valid records spanned the full scale from level 1 to level 9, indicating that the panel included materials ranging from relatively resistant to highly susceptible. The frequency distribution was skewed toward the lower disease scores, with 186, 130, 103, 55, and 14 hybrids assigned to levels 1, 3, 5, 7, and 9, respectively (Figure 2B). These classes represented 38.1%, 26.6%, 21.1%, 11.3%, and 2.9% of the scored panel, indicating that resistant or moderately resistant entries were more common than highly susceptible genotypes. These data indicate a broad phenotypic range across the panel, consistent with quantitative variation in NCLB severity. The broad-sense heritability estimate of 0.6588 (Figure 2C) further indicated a substantial genetic contribution to disease variation while still leaving room for environmental influence, consistent with previous reports describing NCLB resistance as a quantitative trait with moderate to relatively high heritability [5,6,12].

### 2.3. Consensus Association Loci Constitute the Genetic Basis Conferring Resistance to NCLB

**Figure 3:**
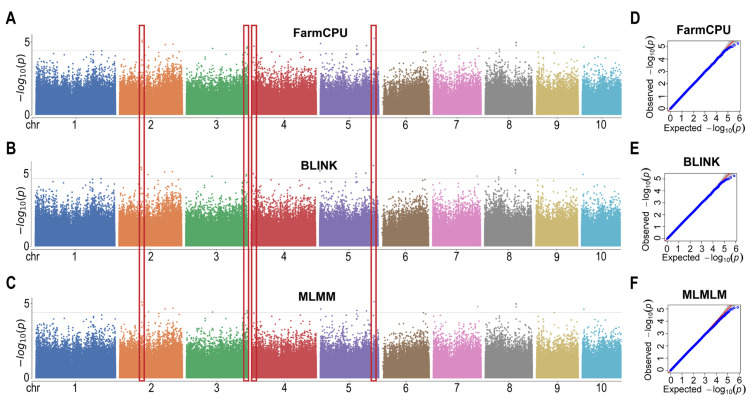
Seventeen consensus association signals for NCLB severity were consistently recovered across three GWAS models. Manhattan plots from FarmCPU (**A**), BLINK (**B**), and MLMM (**C**) revealed a shared set of association peaks, with the most prominent signals located on chromosomes 2, 3, 4, and 5. The corresponding QQ plots were shown on the right (**D**–**F**). The horizontal dashed line denotes the suggestive threshold (−log10(P) = 4.40; P = 3.98 × 10^−5^), and the red boxes highlight four strong loci.

Multi-model GWAS revealed a consistent association landscape for NCLB resistance in the commercial hybrid panel. FarmCPU, BLINK, and MLMM each detected signals above the suggestive threshold of −log10(P) = 4.40 (Figure 3A–C). QQ plots revealed that false-positive inflation caused by the population structure and genetic relatedness was properly controlled (Figure 3D–F).

FarmCPU and BLINK detected 22 significant SNPs, while MLMM detected 17 significant SNPs, all of which were included among the 22 SNPs detected by FarmCPU/BLINK. After merging SNPs within the same LD-supported regions, 17 association loci were retained. Among the four prioritized loci, three exceeded the threshold in all three models, whereas the chr4 locus was significant in two models and showed a near-threshold association signal in MLMM. Therefore, this locus was retained as a relatively stable candidate signal (Supplementary Table S2). The four consensus peaks were located at chr2:88044793, chr3:234450746, chr4:8476302, and chr5:208807210 (Figure 3A–C). These results identify a robust set of candidate loci for downstream experimental validation.

### 2.4. Key Candidate Genes Serve as the Targets for Maize Resistance Breeding

**Figure 4:**
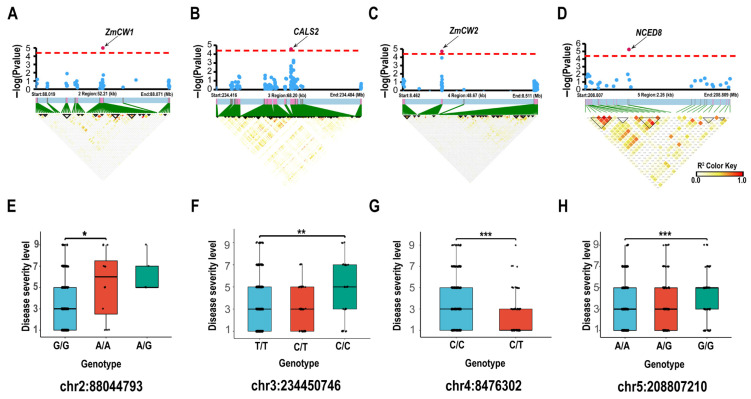
Four prioritized loci were supported by local association patterns and showed clear genotype-dependent differences in NCLB severity. (**A**–**D**) Regional plots around the lead SNPs on chromosomes 2, 3, 4, and 5 revealed clustered association signals within the surrounding linkage LD intervals, thereby narrowing the candidate regions. Using the genome-wide LD-decay estimate of 84.9 kb on either side of each lead SNP, four genes—*ZmCW1*, *CALS2*, *ZmCW2*, and *NCED8*—were retained as positional candidates. (**E**–**H**) Single-locus genotype comparisons were performed using the lead SNPs at these four loci. Asterisks denote significant pairwise differences after Benjamini–Hochberg correction (*, *p* < 0.05; **, *p* < 0.01; ***, *p* < 0.001).

Regional association analysis narrowed the candidate intervals around the four prioritized peak SNPs (Figure 4A–D). Using an 84.9 kb LD-decay window on either side of each lead SNP, we found four annotated protein-coding genes in the Zm-B73-REFERENCE-NAM-5.0 assembly: *ZmCW1*, *CALS2*, *ZMCW2*, and *NCED8*. According to the B73 Zm00001eb.1 annotation, *ZmCW1* encodes the SNARE-interacting protein KEULE, *ZmCW2* encodes a DUF629 domain-containing protein, and *NCED8* encodes carotenoid cleavage dioxygenase 4, whereas *CALS2* is currently annotated as a protein-coding gene with limited functional description. These annotations point to processes that may be relevant to disease response, including vesicle trafficking, stress-related signaling, and organelle-associated regulation [15,16,17,18], indicating these genes may be related to resistance to NCLB disease. Based on the genotypes of the lead SNPs at these four loci (Supplementary Table S2), single-locus genotype comparisons showed significant differences in NCLB severity among genotype classes at all four prioritized SNPs (Figure 4E–H). Collectively, these results highlight several candidate genes and linked variants for future marker development and functional validation in commercial maize hybrids.

To further examine the functional context of the candidate genes, we constructed a candidate-centered network using the maize multi-omics integrative map [19]. *ZmCW1*, *CALS2*, and *ZmCW2* showed 8, 17, and 2 direct connections, respectively, whereas *NCED8* was connected to fewer neighboring genes. The corresponding edge list and weights are provided in Supplementary Table S3. These results suggest that the first three genes occupy relatively more connected positions in the retrieved network. GO enrichment of the network gene set highlighted several terms related to oxidative stress and redox-related processes, and broad intracellular components. In the biological process category, the most informative enriched terms included response to oxidative stress (GO:0006979), oxidation-reduction process (GO:0055114), response to inorganic substance (GO:0010035), single-organism cellular process (GO:0044763), and cellular developmental process (GO:0048869). In the cellular component category, the more specific enriched terms included intracellular (GO:0005622), cytosol (GO:0005829), and intracellular part (GO:0044424), together with the broader enriched terms cell (GO:0005623) and cell part (GO:0044464) (Figure S1). Overall, the enrichment results suggest that oxidative stress, redox-related processes, and broad intracellular components may be associated with the candidate-centered network.

## 3. Discussion

Commercial hybrids represent the final breeding product under actual production conditions. Our panel of 500 commercial hybrids reflects the realistic NCLB resistance level of modern maize production, which cannot be reflected by inbred or experimental mapping populations. The hybrid panel carries a heterozygous genetic background that captures both additive and dominance effects underlying disease resistance [20], whereas inbred lines only reflect additive effects. Unlike loci from inbred-based GWAS, which may perform differently in F_1_ hybrids, our signals are anchored in elite heterozygous backgrounds and can be immediately used for marker-assisted selection.

Although applying a fixed LD window of ±84.9 kb across the whole genome is somewhat arbitrary, owing to LD decay patterns varying substantially along chromosomes and among genomic regions, the relatively short LD-decay distance indicates that this hybrid panel retains fine-mapping resolution despite its structured background. In practical terms, this makes the population suitable for quantitative locus discovery while preserving stronger relevance to deployed germplasm than inbred-based panels [11,13,14].

The observed phenotypic range and the H^2^ estimate of 0.6588 indicate that NCLB severity in this panel contains a substantial genetic component, although environmental effects remain important. Pathogen race composition, disease onset, temperature, and humidity may all affect resistance expression and the estimation under field conditions [1,6]. This finding is consistent with previous reports describing NCLB resistance as a quantitative trait with moderate to relatively high heritability [5,6,12]. However, comparison between this study and previous studies did not find consistent loci. This may be related to the following facts: (1) This study uses commercial hybrid lines and all previous studies use inbred lines; (2) all these studies apply stringent significance thresholds, producing a limited number of significant loci.

The four highlighted candidate genes point to several biologically plausible processes. *ZmCW1* encodes the SNARE-interacting protein KEULE and is a plausible disease-resistance candidate because vesicle trafficking is central to immune secretion, and this locus has also been implicated by GWAS as a candidate region for maize *Ustilago maydis* resistance [15]. *CALS2* encodes a callose synthase-related protein; because callose deposition is an important structural defense response, variation at this locus may affect cell-wall-associated resistance capacity [16]. *ZmCW2* remains poorly characterized in current annotation, but it was identified by genome-wide association and host-by-pathogen prediction analyses as a candidate associated with maize disease progression, making it a reasonable cross-study disease-response candidate for follow-up validation [21]. *NCED8* encodes a nine-cis-epoxycarotenoid dioxygenase, a key rate-limiting enzyme in ABA biosynthesis and stress-induced signaling pathways, linking this locus to stress- and hormone-signaling pathways that may influence disease progression [17,18]. However, the inferred gene functions were mainly derived from homologous gene annotations and expression correlations. Further experimental validation is required for their responses to NCLB infection under pathogen induction, haplotype analysis across diverse breeding materials, and verification using biparental populations or gene-editing materials.

A major strength of this study is that we combined natural-infection phenotyping, population-genetic characterization, multi-model GWAS, and genotype comparison within the same commercial hybrid panel. This design strengthens the connection between association discovery and breeding application. The network-based follow-up analyses provided additional context for the prioritized loci but should be interpreted cautiously. The relatively connected positions of *ZmCW1*, *CALS2*, and *ZmCW2*, together with the enrichment of oxidative stress, redox-related processes, and intracellular-component terms, suggest that these processes may be relevant to NCLB-associated variation. Although the gene network and GO enrichment analyses are predictive, these candidate genes and GO terms correspond to key defense pathways, including phenylpropanoid metabolism, reactive oxygen species homeostasis, cell wall reinforcement, and hormone-mediated defense signaling [22,23], which are critical for plant resistance to biotic stress. They only suggest potential functional connections and regulatory modules, and do not constitute direct evidence for molecular mechanisms underlying disease resistance.

A notable limitation of this study is the use of a single growing season for field trials, which may restrict the generalizability of the identified association signals and candidate loci. The environmental conditions (such as pathogen race variability, humidity, and temperature) have a significant impact on disease severity, and can significantly influence phenotypic expression and genetic effects [24], leading to genotype-by-environment interactions that were not captured in this study. Future studies will focus on multi-environment and multi-year trials to validate the identified loci, further enhancing the translational value of the findings for maize breeding.

## 4. Materials and Methods

### 4.1. Plant Materials and Field Trial

A panel of 500 diverse commercial maize hybrids, collected from commercial companies in China, was used to investigate the genetic basis of resistance to NCLB. Field evaluation was conducted in 2025 at Xinzhou (112.7335° E, 38.4177° N), Shanxi Province, China, under natural NCLB infection. The trial was arranged as an augmented block design. To reduce and adjust environmental variance, we planted 40 test hybrids and the three repeated checks (Zhengdan 958, Xianyu 335, and MY73) within each block, applied standard agronomic management, and did not use artificial inoculation or fungicide treatment. Each plot consisted of four 2 m rows with 60 cm row spacing and 25 cm within-row spacing. Two kernels were sown per hill, and thinned to one plant after emergence. NCLB severity was assessed once a week before maturity. Twelve hybrids were excluded from phenotypic analysis because lodging prevented reliable scoring, leaving 488 valid phenotypic records.

On the basis of the percentage of diseased leaf area, NCLB severity was classified into five grades (1, 3, 5, 7, and 9), corresponding to 0–5%, 6–10%, 11–30%, 30–70%, and 71–100% diseased leaf area, respectively. Categorical scores represent a validated ordinal scale for disease severity and are consistent with previous disease evaluation studies [3]. Disease scores were assigned at the plot level and were treated as numeric phenotypes in downstream analyses. These scores were used as field-based estimates of NCLB severity for subsequent statistical and association analyses. Broad-sense heritability (H^2^) was estimated as H^2^ = (σ^2^P − σ^2^E)/σ^2^P, where σ^2^P denotes the total phenotypic variance among the 488 scored hybrids, and σ^2^E denotes the environmental variance estimated from the repeated check hybrids distributed every 40 plots in the augmented design.

### 4.2. Genotyping and Quality Control

DNA from all 500 hybrids was extracted from kernel samples and genotyped using the MaizeSNP600K liquid-phase array [25]. SNP quality control retained markers with a missing rate below 5% and a minor allele frequency above 0.05. After filtering, 742,600 SNPs remained for population-structure analysis, LD-decay estimation, and GWAS. SNP filtering and LD-related calculations were performed in PLINK [26].

### 4.3. Population Structure, Phylogenetic Relationship, PCA, and LD Decay Analysis

Population structure was analyzed with ADMIXTURE using the filtered SNP dataset. Multiple K values were tested, and cross-validation errors were calculated to determine the optimal number of subpopulations [27]. According to the cross-validation error curve and the elbow criterion, K = 4 was selected as the optimal grouping, and the population-structure results for K = 3, 4, and 5 were also examined. A neighbor-joining phylogenetic tree was constructed [28] to evaluate genetic relationships among the 500 hybrids. Principal component analysis (PCA) was performed with the genome-wide SNP dataset to calculate the proportion of variance explained by each principal component and to generate both the variance-explained plot and the PCA scatter plot. Genome-wide LD decay was calculated with PLINK. According to the elbow criterion, the LD-decay curve reached the plateau region at approximately r^2^ = 0.024 [29], corresponding to an LD-decay distance of 84.9 kb.

### 4.4. GWAS

GWAS for NCLB severity was conducted in GAPIT3 [30] using the FarmCPU [31], BLINK [32], and MLMM [33] models. Combining multiple GWAS models facilitates the identification of consistent loci and the elimination of potential false-positive signals. The analysis included the 488 hybrids with both genotypic and phenotypic data. Principal components and a kinship matrix were incorporated to control for population structure and genetic relatedness. The effective number of SNPs estimated from the genome-wide LD-decay distance was 25,110, and the suggestive threshold was set at −log10(P) = 4.40 (P = 3.98 × 10^−5^). SNPs within the same LD-supported region were merged into a single locus, and the SNP with the lowest *p* value within each locus was designated as the lead SNP. Manhattan and QQ plots were used to assess signal patterns and model fit. To reduce false positives, only loci detected by all three models were retained as consensus signals, and lead SNPs of consensus loci were prioritized for downstream candidate-gene and genotype analyses.

### 4.5. Lead SNP-Based Candidate-Gene and Single-Locus Genotype Analysis

Candidate genes were selected from the annotated genes overlapping the lead SNP or located within 84.9 kb upstream or downstream of the lead SNP. On the basis of this criterion, *Zm00001eb086800* (*ZmCW1*), *Zm00001eb163000* (*CALS2*), *Zm00001eb167550* (*ZmCW2*), and *Zm00001eb251990* (*NCED8*) were retained as candidate genes. Single-locus genotype comparisons were then performed to evaluate differences in NCLB severity among genotype classes at the prioritized SNPs. Pairwise comparisons among genotype classes were conducted using the Wilcoxon rank-sum test [34] followed by Benjamini–Hochberg multiple-testing correction [35].

To further explore the functional context of the prioritized loci, a candidate gene-centered network was constructed by querying the four candidate genes against the maize multi-omics integrative network map [19]. The directly connected genes and corresponding edge weights were extracted for each candidate gene. Candidate genes showing clear network connections, together with their directly connected genes, were submitted to agriGO v2.0 [36] for Gene Ontology (GO) enrichment analysis. Enrichment was summarized across the biological process, cellular component, and molecular function categories, and GO terms with adjusted *p*-values below 0.05 were considered significant.

## 5. Conclusions

This study is the first to conduct GWAS on commercial maize hybrids, confirming the feasibility of using hybrid populations for genetic mapping and filling the gap between theoretical genetic research and practical breeding applications. Combining multiple statistical models effectively reduced false-positive signals, identifying stable association loci that are closely related to maize NCLB resistance. Four candidate genes were supported by functional annotation and expression analysis, providing direct targets for molecular breeding. However, the study has limitations: phenotypic data were obtained from a single growing environment and natural infection conditions, which may affect the generalizability of the results. Future research will focus on multi-environment, multi-season trials to further verify the stability of the identified loci.

## Data Availability

The original contributions presented in this study are included in the article/Supplementary Material. Further inquiries can be directed to the corresponding author.

## Supplementary Materials

The following supporting information can be downloaded at: https://www.mdpi.com/article/10.3390/ijms27114983/s1.
