# Supplementary material for: Genetic Dissection of Resistance to Northern Corn Leaf Blight in a Large Commercial Maize Hybrid Population
Source: Int J Mol Sci. 2026 May 30;27(11):4983. doi: 10.3390/ijms27114983 (PMC13256691; doi:10.3390/ijms27114983)
Supplement: Supplementary file 1 [file ijms-27-04983-s001.zip › Supplementary materialls.pdf]

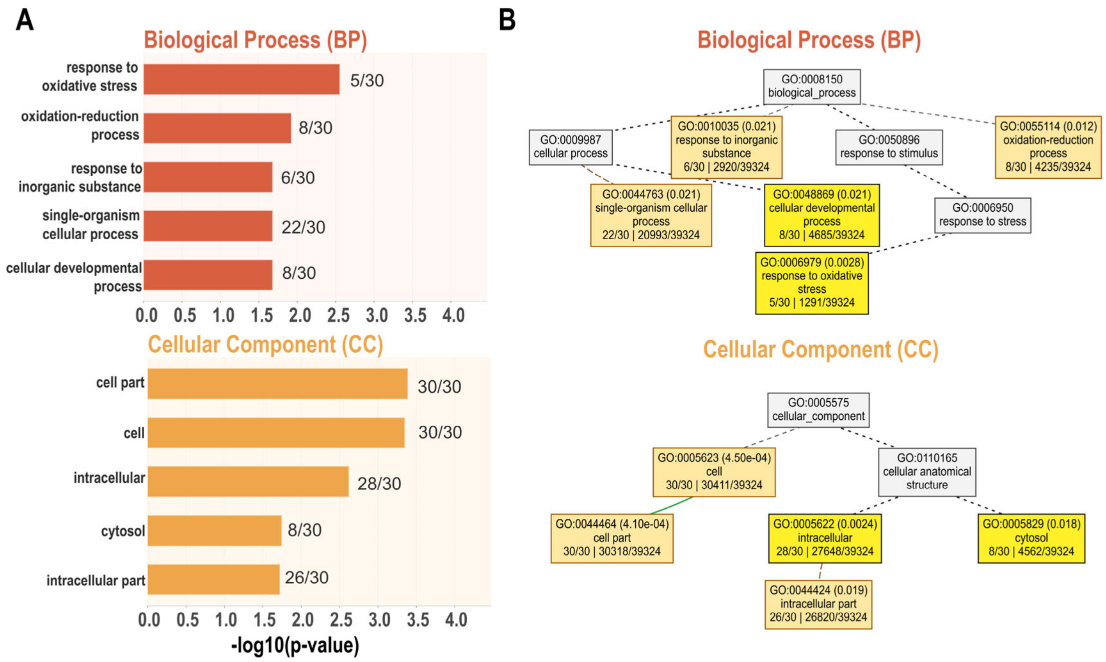

Supplementary Figure 1 GO enrichment analysis of the network-derived gene set associated with the prioritized candidate genes. (A) The enriched biological process terms were dominated by oxidative-stress- and redox-related processes, particularly response to oxidative stress (GO:0006979), oxidation-reduction process (GO:0055114), response to inorganic substance (GO:0010035), single-organism cellular process (GO:0044763), and cellular developmental process (GO:0048869), whereas the cellular component category highlighted cell (GO:0005623), cell part (GO:0044464), intracellular (GO:0005622), cytosol (GO:0005829), and intracellular part (GO:0044424). (B) Across GO categories, the enrichment network further pointed to broad intracellular and cytosolic cellular components together with oxidative-stress, oxidation-reduction, response-to-inorganic-substance, and developmental-process branches. Only terms with adjusted P values $\leqslant 0.05$ are shown.

1 Supplementary Tables

Supplementary table 1 Subgroup assignment, admixture value, and NCLB phenotype of each coded hybrid

Supplementary table 2 Consensus signals identified by three GWAS models

Supplementary table 3 Edge list and connection weights in the candidate-centered co-expression network
